# Human coronavirus OC43 infection associated pneumonia in a girl with acute lymphoblastic leukemia: A case report

**DOI:** 10.1097/MD.0000000000021520

**Published:** 2020-08-14

**Authors:** Tsung-Yen Chang, Chia-Jui Du, Chih-Chen Chang, Shih-Hsiang Chen, Chih-Jung Chen, Chih-Yung Chiu, Cheng-Hsun Chiu

**Affiliations:** a Division of Pediatric Hematology-Oncology; b Division of Pediatric General Medicine; c Department of Medical Imaging and Intervention; d Division of Pediatric Infectious Diseases; e Division of Pediatric Pulmonology, Chang Gung Memorial Hospital, College of Medicine, Chang Gung University, Taoyuan, Taiwan.

**Keywords:** acute lymphoblastic leukemia, children, febrile neutropenia, human coronavirus OC43, pneumonia

## Abstract

**Rationale:**

Information regarding the clinical features and outcomes of pneumonia due to an infection with human coronavirus (HCoV)-OC43 in children with cancer is rare. This report presents the clinical features in terms of chest CT scan images which may be used to identify cases of HCoV-OC43 infection induced pneumonia in immunocompromised children.

**Patient concerns:**

We report here a girl with acute lymphoblastic leukemia who developed respiratory symptoms during febrile neutropenia. Rapid clinical progression and nodular lesions on her chest X-ray and computed tomography scans were suggestive of a pulmonary fungal infection.

**Diagnosis:**

A series of tests eventually confirmed the exclusive presence of HCoV-OC43 by the FilmArray Respiratory Panel from a throat swab sample.

**Interventions:**

After the diagnosis was confirmed, the antimicrobial agents initially administered were discontinued.

**Outcomes:**

Although the chest CT scan images looked severe, the clinical course of the infection induced pneumonia was benign. The respiratory status of the patient was completely resolved in 2 weeks.

**Lessons:**

This report highlights the importance of early identification of respiratory viruses, via the realization of their clinical characteristics, which helps reduce the duration of administration of antimicrobial agents in this setting.

## 1 Introduction

The human coronavirus (HCoV)-OC43 is one of the common coronaviruses to cause an infection in healthy children, and usually induces only mild upper respiratory diseases. Approximately 30% to 33% of children with HCoV-OC43 infection have been diagnosed with lower respiratory tract infection.^[1,2]^ Despite this, information regarding the clinical features and outcomes of an HCoV-OC43 infection in children with cancer is limited. In this report, we present a young girl with acute lymphoblastic leukemia (ALL) who developed pneumonia resulting from an HCoV-OC43 infection, which was characterized by severe chest CT scan images but a benign clinical course.

### 1.1 Patient presentation

**Figure 1:**
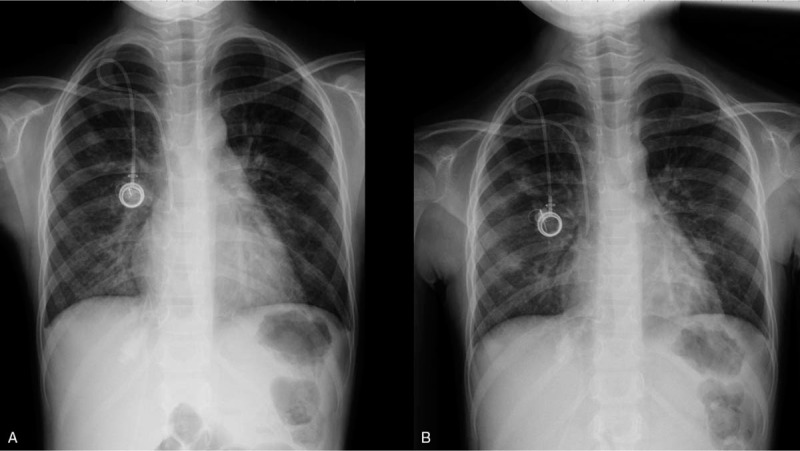
A: Increased infiltration in right lower lobe. B: Patchy nodular opacities in right middle lobe and bilateral lower lobes.

**Figure 2:**
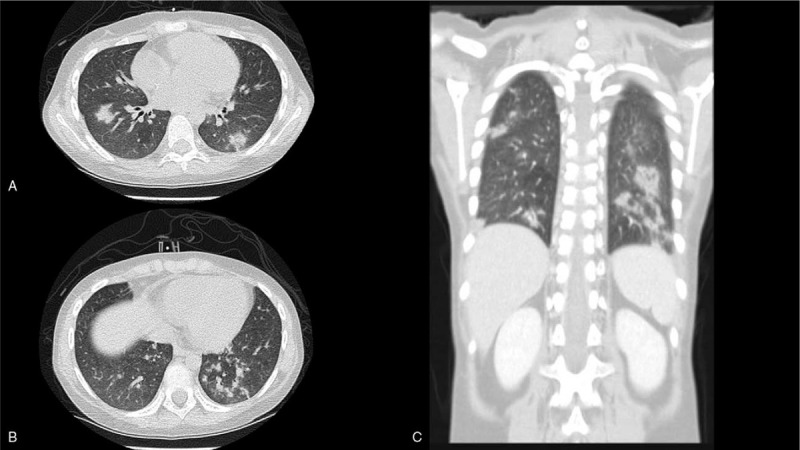
Multiple ill-defined nodular and wedge-shape opacities in bilateral lung fields (A & B), predominantly in bilateral lower lobes (C).

An 8-year-old girl presented with fever and an abnormal hemogram (leukocyte count 29.7 × 10^9^ cells/L with 49% blasts, hemoglobin 7.9 g/dl, and platelet count 231 × 10^9^ cells/L). She had been diagnosed with B cell precursor ALL on December 24th, 2019. Cytogenetic analysis disclosed the presence of the karyotype 46,XX,del(9)(q34),t(12;22)(p13;q11.2), while molecular analysis was negative for *ETV6-RUNX1*, *TCF3-PBX1*, and *BCR-ABL1*. The girl received chemotherapy according to the Taiwan Pediatric Oncology Group (TPOG) 2013-ALL-SR protocol, and spinal tap and triple intrathecal therapy was performed 7 to 10 days after chemotherapy.^[3]^ No leukemic cells were found in the cerebral spinal fluid and the patient commenced prophylactic antibiotic and antifungal therapies with levofloxacin and micafungin on January 14th, when her leukocyte count was less than 1 × 10^9^ cells/μl. She later developed febrile neutropenia on January 20th, and was administered teicoplanin and meropenem post collection of her blood for culture. Her blood culture yielded *Streptococcus mitis* which is sensitive to teicoplanin. However, she gradually developed a cough in parallel with a respiratory tract infection from her mother who was taking care of her in the hospital. A chest X-ray on January 22nd showed increased infiltration in the right lower lobe (Fig. 1A). In the meantime, the optical density index (ODI) of serum galactomannan was 0.07. She was then administered with azithromycin for 3 days as atypical pneumonia resulting from a bacterial infection was suspected. Despite this, the patients fever was not resolved and her cough further worsened even after her leukocyte count increased (1.6 × 10^9^ cells/μl with granulocytes 0.08 × 10^9^ cells /μl and lymphocytes 0.85 × 10^9^ cells /μl on January 25). She had increased difficulty in breathing requiring low-flow nasal cannula support and auscultation yielded a coarse breathing sound without wheezing or rales. A second chest X-ray revealed patchy nodular opacities in the right middle lobe and bilateral lower lobes (Fig. 1B). Further, computed tomography (CT) of the chest showed multiple ill-defined nodular and wedge-shape opacities predominantly in the bilateral lower lobes (Fig. 2). Since pulmonary fungal infection was initially suspected, liposomal amphotericin-B was substituted for micafungin. Concurrently, the ODI of serum galactomannan increased to 0.15. Finally, a throat swab sample was collected and tested using the FilmArray Respiratory Panel, a multiplex polymerase chain reaction (PCR) assay that can detect up to 16 viruses and the 3 bacterial targets, *Mycoplasma pneumoniae*, *Bordetella pertussis,* and *Chlamydia pneumoniae*. The result of this assay confirmed the exclusive presence of HCoV-OC43. Although the cough gradually improved, the parents agreed to let the patient undergo bronchoscopy with collection of bronchoalveolar lavage (BAL) fluid 1 week later. The BAL samples were subjected to multiple staining and culture, including Gram staining and special stains in cytology, which later revealed the samples to be negative for fungi, acid-fast bacilli, and viruses. Thus, antimicrobial agents were discontinued. Her respiratory status became asymptomatic in the following week, and a chest X-ray 1 month later showed complete resolution of the pulmonary infiltrates.

Informed written consent was obtained from the mother of the patient for publication of this case report and any accompanying images.

## 2 Discussion

Before the outbreak of the coronavirus disease of 2019 (COVID-19), 6 types of human coronaviruse (HCoV) have been known to cause infections, namely HCoV-OC43, HCoV-299E, HCoV-HKU1, HCoV-NL63, severe acute respiratory syndrome (SARS)-CoV, and middle east respiratory syndrome (MERS)-CoV. It has been reported that the HCoV-OC43 infection occurs frequently in early childhood and largely presents symptoms of upper respiratory tract infection.^[4]^ Pneumonia caused by HCoV-OC43 infection is seldom discussed, especially in children with cancer.

Although HCoV-OC43 is predominantly associated with upper respiratory tract infections, there have been cases where it has been associated with a serious infection in immunocompromised children. Simon et al reported a 5-year-old boy with ALL who presented with febrile neutropenia and pneumonia.^[5]^ Real-time polymerase chain reaction assay of the nasopharyngeal aspirate sample confirmed an HCoV-OC43 infection, and after the leukocyte count increased, the respiratory condition of the young boy reportedly improved. In another study, Morfopoulou et al reported an 11-month-old boy with severe combined immunodeficiency who contracted HCoV-OC43-associated encephalitis after he underwent cord blood transplantation and died 1.5 months after the transplantation.^[6]^ In yet another study, Nilsson et al reported a 9-month-old infant with ALL who had persistent HCoV-OC43-associated respiratory infection and developed fatal encephalitis.^[7]^ Therefore, it is of paramount importance that physicians be aware of the risk of the serious complications associated with the HCoV-OC43 infection during the treatment of immunocompromised children.

Extensive or multifocal ground glass opacification with consolidations is a common sign in chest radiography, which is suggestive of pneumonia caused by the highly pathogenic coronaviruses, SARS-CoV, MERS-CoV, or COVID-19.^[8,9]^ However, little is known about the radiographic findings of pneumonia caused by the other coronaviruses, which generally cause self-limited upper respiratory tract infections in immunocompetent patients. Pene et al reported 2 immunocompromised patients with pneumonia caused by HCoV-299E infection, of whom one had chest CT images that showed multiple micronodular opacities.^[10]^ In this report, our patients chest CT scan revealed multiple nodular and wedge-shape opacities, predominantly in the bilateral lower lobes and the right upper lobe, which were similar to those reported by Pene et al To the best of our knowledge, our report is the first to describe the specific features of a chest CT scan in pediatric cancer patients with HCoV-OC43 pneumonia.

Febrile neutropenia is a common complication of cancer treatment in children. Because of the decreased inflammatory response, bacterial and fungal infections in neutropenic patients are the major causes of morbidity and mortality. As a result, there is an updated guideline, which focuses on strategies for antibacterial and antifungal therapies for the management of febrile neutropenia in children with cancer or hematopoietic stem cell transplantation.^[11]^ Recently, there has been a growing emphasis on the identification of viral infections to enable the application of individualized treatments and reduce the antibiotic burden in children with cancer and febrile neutropenia.^[12–14]^ Although we identified HCoV-OC43 from the throat swab sample, we modified and continued the administration of antimicrobial agents to our patient due to the severe chest images we obtained that were mimicking serious pulmonary infections. Nonetheless, rapid improvement of the respiratory status and negative microbiological results on the BAL fluid sample were suggestive of a benign course of the HCoV-OC43 infection-associated pneumonia. Therefore, it is important for physicians to recognize the clinical characteristics of such an infection so that the duration of the antimicrobial therapy may be reduced.

In conclusion, our report describes the case of pneumonia caused due to an HCoV-OC43 infection during the induction therapy of a girl with ALL. Although HCoV-OC43 infection occasionally causes serious lower respiratory infection in immunocompromised children, as presented by severe chest images, its clinical course may be benign as is the case with our patient.

## Author contributions

**Conceptualization:** Shih-Hsiang Chen, Tsung-Yen Chang, Chia-Jui Du.

**Data curation:** Shih-Hsiang Chen, Tsung-Yen Chang, Chia-Jui Du.

**Formal analysis:** Shih-Hsiang Chen.

**Investigation:** Chih-Chen Chang, Chih-Jung Chen, Chih-Yung Chiu.

**Supervision:** Cheng-Hsun Chiu.

**Validation:** Chih-Chen Chang, Chih-Jung Chen, Chih-Yung Chiu.

**Visualization:** Chih-Chen Chang, Chih-Jung Chen, Chih-Yung Chiu.

**Writing – original draft:** Tsung-Yen Chang, Chia-Jui Du, Shih-Hsiang Chen

**Writing – review & editing:** Shih-Hsiang Chen, Cheng-Hsun Chiu.
